# A Retrospective Analysis of Vinorelbine Chemotherapy for Patients With Previously Treated Soft-Tissue Sarcomas

**DOI:** 10.1155/SRCM/2006/15947

**Published:** 2006-11-21

**Authors:** Sibyl E. Anderson, Mary L. Keohan, David R. D'Adamo, Robert G. Maki

**Affiliations:** ^1^Gynecological Oncology Services, Department of Medicine, Memorial Sloan-Kettering Cancer Center, New York, NY 10021, USA; ^2^Melanoma-Sarcoma Services, Department of Medicine, Memorial Sloan-Kettering Cancer Center, New York, NY 10021, USA

## Abstract

*Introduction*. The role of vinorelbine in specific soft tissue sarcoma subtypes is unclear. We present retrospective single institution experience with single-agent vinorelbine in subjects with metastatic soft tissue malignancies. *Methods*. Fifty-eight patients were treated with single agent intravenous vinorelbine between April 1997 and December 2004. Doxorubicin had been administered previously to 53 subjects (91%), and the median number of lines of previous chemotherapy was 3 (range 0–7). *Results*. Patients received a median 6 doses of vinorelbine (range 1–65). The overall response rate was 6% (3 patients: 1 angiosarcoma, 1 epithelioid sarcoma, and 1 embryonal rhabdomyosarcoma). Fourteen patients (26%) experienced a best result of stable disease. Median time to progression was 1.8 months (95% confidence intervals 1.5–2.1 months, Kaplan-Meier estimate). Eight patients experienced grade 3 or 4 toxicity, most commonly febrile neutropenia. *Conclusion*. Vinorelbine demonstrates limited activity in a heavily pretreated group of soft-tissue sarcoma patients. Prospective investigation may be considered for selected sarcoma subtypes.

## INTRODUCTION

Soft-tissue sarcomas are a large group of rare and heterogeneous cancers from the extraskeletal connective tissues that make up less than 1% of adult malignancies. Estimated incidence of soft tissue sarcoma is 9530 new cases in the United States in 2006, an incidence rate of approximately 32 per million [1]. Soft tissue sarcoma may originate in any part of the body and metastasize primarily to the lungs and also to bone, liver, and other organs depending on the subtype. The median survival for patients diagnosed with metastatic or recurrent unresectable sarcoma is usually less than 12 months although a limited number of patients may have long term survival as the result of optimal response to chemotherapy [2]. Doxorubicin remains the most active drug in the treatment of soft tissue sarcoma with a response rate of 10–25% in previously untreated patients [2]. Other active drugs or combinations include ifosfamide [3], gemcitabine with or without docetaxel [4, 5], and dacarbazine (DTIC) [6]. Treatment options are therefore limited and responses are often of short duration.

It is increasingly appreciated that each sarcoma subtype has specific patterns of sensitivity to standard chemotherapy agents. For example, leiomyosarcomas are often relatively resistant to ifosfamide [2], and sensitive to dacarbazine [5], compared to other sarcoma subtypes, and angiosarcomas are relatively unique in their response to taxanes. Furthermore, clinical trials involving metastatic sarcomas allow only limited lines of chemotherapy as an entry criterion, leaving heavily treated subjects without well-examined options among commercially available cytotoxic agents. There are reports of responses of soft-tissue sarcoma patients to vinorelbine [7–9]. However, the role of vinorelbine in specific adult soft-tissue sarcoma subtypes is unclear. We present here retrospective single institution experience with vinorelbine in selected soft-tissue sarcoma patients, many of whom were heavily pretreated, in the hope of identifying subtypes of sarcoma meriting prospective evaluation of this relatively nontoxic agent.

## METHODS

After Institutional Review Board (IRB) approval was obtained, we reviewed our database of patients with recurrent or metastatic unresectable leiomyosarcoma treated by Memorial Sloan-Kettering Cancer Center's (MSKCC) Adult Medical Oncology Service with vinorelbine between April, 1997 and December, 2004. Patients were treated with cycles of vinorelbine chemotherapy consisting of 15 to 30 mg/m^2^ weekly for 2 or 3 weeks, followed by 1 week rest. Patients' charts were reviewed for age at time of vinorelbine therapy, stage, performance status, prior chemotherapy treatment, vinorelbine dose and schedule, best response to treatment, and treatment delays or dose reductions due to toxicity. Responses were evaluated via CT scan or MRI, and assessed according to response evaluation criteria in solid tumors (RECIST) by radiologists at our institution. Overall and progression-free survival curves were constructed and Kaplan-Meier estimates were generated using SPSS version 12.0. We also performed a review of the literature pertaining to vinorelbine in sarcomas.

## RESULTS

Patient characteristics are indicated in Table 1. We identified 66 patients with soft-tissue sarcomas treated at Memorial Hospital between April 1997 and December 2004. Five patients with GIST and three patients with deep fibromatoses (desmoid tumors) treated with vinorelbine were excluded, leaving 58 patients, who form the group analyzed here. (No patients with GIST responded; the best result for desmoid tumor was stable disease.) There were 26 men and 32 women in the remaining group. The median age was 52 years (range 20–76 years). Forty-three patients (74%) had metastatic disease to lungs and 20 (34%) patients' metastases involved liver. Fifty of the tumors were termed high-grade; and seven low-grade; for one a determination of grade could not be made. Three patients had radiation-induced sarcomas. The median Karnofsky performance status was 80% (range 60–100%). The median number of previous chemotherapy regimens was 3 (range 0–7), and median number of different chemotherapy drugs received was also 3 (range 0–14). Fifty-three subjects (91%) of patients had received prior doxorubicin-based chemotherapy.

### Vinorelbine therapy and clinical and radiological responses

The median starting dose of vinorelbine in this retrospective analysis was 25 mg/m^2^ per dose (range 15–33 mg/m^2^). The median number of doses administered was 6 (range 1–65). Eleven patients required a dose reduction; one patient received two dose reductions over the course of her therapy.

**Figure 1:**
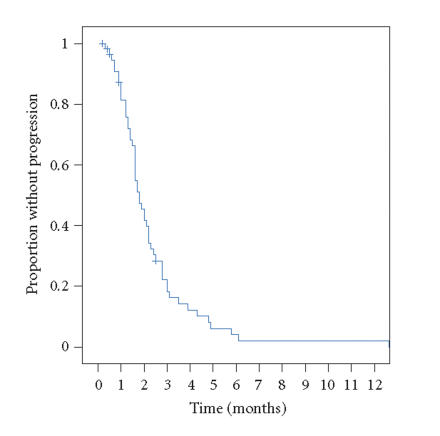
Progression-free survival curve for patients receiving vinorelbine on this study (+ indicates censored patient).

**Figure 2:**
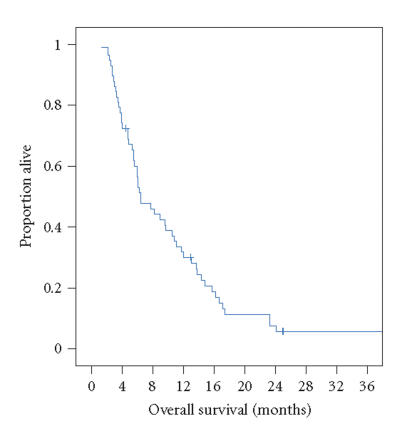
Overall survival for patients receiving vinorelbine on this study (+ indicates censored patient).

Of the 58 patients examined, 3 patients (angiosarcoma, epithelioid sarcoma, and embryonal rhabdomyosarcoma) experienced a partial response, and received vinorelbine for 3.0, 27.4, and 4.8 months, respectively. No complete responses were observed. Two patients had minor responses (angiosarcoma, MFH (malignant fibrous histiocytoma, now termed high-grade undifferentiated pleomorphic sarcoma)). Fourteen patients (26%) demonstrated stable disease as best response, five patients experienced stable disease for 3 months or more and only one patient for more than 6 months. The Kaplan-Meier estimate for median time to progression for the entire cohort was 1.8 months (95% confidence intervals 1.5–2.1 months), see Figure 1. The Kaplan-Meier median overall survival estimate was 6.4 months (95% confidence intervals 4.1–8.7 months), see Figure 2.

## TOXICITY

The primary toxicity was hematologic. The severity of toxicity did not appear to be related to dose or schedule. No deaths were attributed to drug. No patients received prophylactic filgrastim or pegfilgrastim. A total of 8 patients (14%) experienced grade 3-4 toxicities that could be possibly, probably, or definitely related to vinorelbine administration. One patient had ∼5 cm^2^ extravasation injury from vinorelbine, given in a peripheral vein (grade 3 toxicity). Three patients experienced febrile neutropenia (grade 3) after vinorelbine chemotherapy. One patient was hospitalized for grade 4 mucositis, one for pulmonary embolus (possibly related), one for dehydration, nausea, and vomiting, and one for grade 3 obstipation requiring hospitalization.

## DISCUSSION

Soft-tissue sarcomas are rare tumors for which salvage therapies yield the unfortunate combination of short response duration and relatively great toxicity. Doxorubicin has been the standard chemotherapeutic intervention for 30 years, and remains the most effective single agent [2]. The role and activity of other salvage agents is becoming better defined as we learn which subtypes of sarcoma respond best to different cytotoxic agents, a reflection of more consistent pathology review, better imaging, and a larger armamentarium of chemotherapy agents to test. Vinorelbine is a well-tolerated semisynthetic vinca alkaloid extracted from *Vinca rosea*, inhibiting microtubule assembly. Initial phase I and phase II trials revealed neutropenia and neuropathy as its most common toxicities. In the present study, febrile neutropenia was the most frequent significant adverse event associated with vinorelbine chemotherapy, and reflects not only the known toxicities of vinorelbine but also the cumulative prior therapy received by individual patients.

Pediatric sarcoma trials suggest a possible salvage role for vinorelbine in selected adult sarcomas [7], as highlighted by the brief response of a patient with embryonal rhabdomyosarcoma in this study. Thirty-three pediatric soft tissue sarcoma patients were treated with weekly vinorelbine: 30 mg/m^2^ in days 1 and 8 of 21-day schedule by the Pediatric Unit of the Istituto Nazionale Tumori in Milan. Twenty-three percent of patients had received 2 or more previous lines of chemotherapy and 18 patients had metastasis to lung. Grade 3 or 4 neutropenia was observed in 27% and 36% of patients, respectively; occurring in 50% of patients who had previously undergone high-dose chemotherapy with stem cell rescue. Of 28 evaluable patients 28% achieved partial response and 9 (32%) achieved stable disease. Impressive activity was noted in rhabdomyosarcoma patients with 7 of 12 patients (58%) achieving partial or major response and 2 patients (8%) with stable disease. One of 5 osteosarcomas (20%) achieved PR and 1 of 7 (14%) Ewing sarcomas achieved PR. Median duration of partial response was 10 months and stable disease 3.5 months. The remaining 10 patients progressed on therapy.

Single-agent vinorelbine also demonstrates activity in AIDS-associated Kaposi sarcoma [8]. Of 35 assessable adult patients treated by the Italian Cooperative Group on AIDS and Tumors with vinorelbine 30 mg/m^2^ every 2 weeks, 34% achieved a partial response and 9% acheived a complete response for a median duration of 176 days. Of note, all patients received prior chemotherapy. Nonhematologic toxicity was uncommon. The most common toxicity observed was neutropenia: grade 4 (30%) and grade 3 (21%) with the majority of patients requiring filgrastim to support their neutrophil counts. Thirty-six severely immune-compromised patients were treated with no toxic-related deaths reported.

Fidias et al performed a phase II trial of weekly vinorelbine at 30 mg/m^2^ in 37 adult sarcoma patients who failed doxorubicin-based therapy [9]. Activity was observed in angiosarcoma and leiomyosarcoma patients. Of 35 evaluable patients 1 demonstrated a complete response and 6 showed partial responses. Three patients demonstrated stable disease. Of 4 patients with angiosarcoma, one demonstrated complete response, and 2 a mixed response.

Given the relative chemoresistance of soft-tissue sarcomas, and patterns of response seen for a variety of sarcomas, stable disease and time to progression may indicate a significant response and therefore may be a more appropriate therapeutic endpoint than response per se. Van Glabbeke and colleagues investigated what a reasonable estimate for progression-free survival at 3 and 6 months are for active and inactive agents by examining the results from prospective clinical trials conducted by the EORTC [10]. The progression-free rates were 39% and 14% at 3 months and 6 months, respectively, for agents that were active, in comparison to 21% and 8% for inactive agents. The progression free rate for vinorelbine in this cohort of patients was only 18% at 3 months and 2% at 6 months, indicative of an inactive agent. However, this may not be a fair comparison, since vinorelbine in this analysis was given in 4th line (median) in comparison to 1st or 2nd line in the EORTC database analysis.

There is an increasing body of evidence supporting the variable response of soft-tissue sarcoma subtypes. Initial salvage combination trials of vinorelbine suggest such combinations have activity in selected adult and pediatric soft-tissue sarcomas. The activity of vinorelbine as a single agent is minor compared to the higher responses rates, time to progression, and overall survival seen for gemcitabine and docetaxel in subjects with leiomyosarcoma [5], MFH [11–13], and pleomorphic liposarcoma [13], and of ET-743 (trabectedin) for patients with myxoid-round cell liposarcoma [14]. Nonetheless, this small retrospective investigation suggests vinorelbine potentially has antitumor activity in specific soft-tissue sarcomas and is relatively well tolerated in heavily pretreated patients. A further prospective analysis in less heavily treated patients with rhabdomyosarcoma, angiosarcoma, and epithelioid sarcoma appears warranted, based on the hints of activity seen in this highly selected population of patients well enough to tolerate multiple lines of therapy.

## Figures and Tables

**Table 1:** Patient demographics.

Total number of subjects	58

Male	26 (45%)
Female	32 (55%)
Median age (range)	52 (20–76)
Subjects receiving prior doxorubicin	53 (91%)
High grade primary sarcoma	50 (88%)
Low grade primary sarcoma	7 (12%)
Unknown	1
Radiation-associated primary tumor	3 (5%)
Lung metastases present	43 (74%)
Liver metastases present	20 (54%)
Median prior lines of therapy (range)	3 (0–7)
Median prior total number of agents administered (range)*	3 (0–14)
Median KPS^@^(range)	80% (60%–100%)
Median number of doses of vinorelbine administered (range)	6 (1–65)
Median starting dose (mg/m^2^) (range)	25 (15–33)
Histology:
Angiosarcoma	7
Cystosarcoma phylloides	1
Desmoplastic small round cell tumor	1
Endometrial stromal sarcoma	3
Epithelioid sarcoma	2
Ewing sarcoma/peripheral neuroectodermal tumor	1
Fibromyxoid sarcoma	1
Fibrosarcoma	1
Leiomyosarcoma	20
Liposarcoma	4
Mesenchymal chondrosarcoma	1
MFH (malignant fibrous histiocytoma)	8
Rhabdomyosarcoma, embryonal	1
Sarcoma, not otherwise specified	3
Synovial sarcoma	4

* Each drug only counted once, except ifosfamide, if given in high doses ≥10 g/m^2^/cycle, which counts as a separate agent in this analysis.

^@^ Karnofsky performance status.
